# HER2-low expression does not affect the clinical outcomes of metastatic breast cancer treated with CDK4/6 inhibitor: A real-world study

**DOI:** 10.3389/fendo.2022.1000704

**Published:** 2022-08-18

**Authors:** Yingbo Shao, Zhifen Luo, Yang Yu, Qi Chen, Yaning He, Chaojun Liu, Bing Nie, Fangyuan Zhu, Hui Liu

**Affiliations:** ^1^ Department of Breast Oncology, Henan Provincial People’s Hospital, Zhengzhou University People’s Hospital, Zhengzhou, China; ^2^ Department of Breast Oncology, Henan Provincial People’s Hospital, Henan University People’s Hospital, Zhengzhou, China; ^3^ Department of Medical Oncology, Henan Provincial People’s Hospital, Zhengzhou University People’s Hospital, Zhengzhou, China; ^4^ Department of Medical Oncology, Henan Provincial People’s Hospital, Henan University People’s Hospital, Zhengzhou, China

**Keywords:** breast cancer, CDK4/6 inhibitor, HER2-low, endocrine therapy, palbociclib

## Abstract

**Background:**

There is accumulating evidence support human epidermal growth factor receptor 2 (*HER2*)-low as a biologically distinct subtype of breast cancer. The present study was conducted to explore whether *HER2*-low expression will affect the clinical efficacy of cyclin-dependent kinase (*CDK*) 4/6 inhibitor for patients with hormone receptor (HR)-positive, *HER-2* negative metastatic breast cancer.

**Methods:**

Patients with HR+/*HER2*- metastatic breast cancer who were treated with palbociclib from January 2019 to June 2021 were retrospectively analyzed based on real-world clinical practice. *HER2*-zero was defined as immunohistochemistry (IHC) 0, and *HER2*-low was defined as IHC 1+ or IHC 2+/fluorescence *in situ* hybridization (FISH) negative. The primary end point was progression free survival (PFS), and the secondary end points were objective response rate (ORR), disease control rate (DCR), overall survival(OS) and safety.

**Results:**

45 patients received palbociclib plus aromatase inhibitor (AI) or fulvestrant therapy, including 24 *HER-2*-zero and 21 *HER-2*-low patients. There were no statistically significant differences in clinicopathological characteristics between the two groups. No significant differences were observed in ORR (41.7% vs. 28.6%, P=0.360) and DCR (79.2% vs. 76.2%, P=0.811) between *HER-2*-zero and *HER-2*-low patients. And simultaneously, *HER2*-zero and *HER2*-low patients obtained similar median PFS (16.2m vs. 14.1m, P=0.263). The median OS was not reached. Neutropenia and leukopenia were the most common adverse events. Grade 3-4 adverse events(AEs) occurred in 58.3% and 57.1% of patients, respectively.

**Conclusions:**

*HER2*-low expression does not affect the clinical efficacy of palbociclib and our present study did not support incorporating *HER2*-low into systemic therapy decisions for patients with HR+/*HER2*- metastatic breast cancer treated with *CDK4/6* inhibitor.

## Introduction

Global cancer registration data in 2020 demonstrated that breast cancer has become the most common malignant tumor in the world, accounting for 11.7% of all cancers (1). Although the overall treatment level of breast cancer has improved significantly in recent years, metastatic breast cancer is still a bottleneck affecting the prognosis of patients. With the success of the PALOMA (2–4), MONARCH (5–7) and MONALEESA (8–10) series of trials, cyclin-dependent kinase (CDK) 4/6 inhibitor plus traditional endocrine therapy has become the standard treatment strategy for hormone receptor (HR)-positive and human epidermal growth factor receptor 2 (*HER2*) negative metastatic breast cancer, which prompting HR+/*HER2*- metastatic breast cancer to enter a new era of targeted therapy combined endocrine therapy.

Many clinical studies have demonstrated that antibody drug conjugate(ADC) drugs are not only effective in *HER2*-positive breast cancer, but also show good anti-tumor activity in patients with low *HER2* expression (11, 12). And there is accumulating evidence support *HER2*-low as a distinct subtype of breast cancer (13). Most clinical trials use immunohistochemistry (IHC) 1+, or IHC 2+ and FISH negative as the definition of *HER2*-low breast cancer and the proportion of people with *HER*2 low expression is as high as 45%-55% with this diagnostic criteria (14, 15). However, there has been little focus on this patient population with low expression of *HER2* due to lack of understanding of its biological behavior and absence of tailored treatment manner.

Our previous research confirmed that *HER2*-low breast cancer exhibit specific clinicopathological features and different response to neoadjuvant chemotherapy (NAC). However, there have been no reports on whether the low expression of *HER2* is related to the treatment response of endocrine therapy for metastatic breast cancer. The present study was conducted to explore whether *HER2*-low expression will affect the clinical efficacy of endocrine therapy for patients with HR+/*HER2*- metastatic breast cancer who received *CDK4/6* inhibitor.

## Methods

### Patient population

From January 2019 to June 2021, patients with HR+/*HER2*- metastatic breast cancer who have received palbociclib plus aromatase inhibitor (AI) or fulvestrant therapy at Henan Provincial People’s Hospital were retrospectively collected in this study. The main selection criteria included: 1) histopathological confirmed metastatic breast cancer; 2) HR+/*HER2* negative, which determined by immunohistochemistry (IHC) or fluorescence *in situ* hybridization (FISH); 3) have received palbociclib plus AI or fulvestrant therapy; 4) based on RECIST v1.1, presence of at least one measurable lesion. The exclusion criteria included: 1) received less than two cycles of palbociclib plus AI or fulvestrant treatment and could not evaluate the efficacy; 2) follow-up data are not available.

### Treatment and follow-up

Estrogen receptor (ER) and progesterone receptor (PR) were detected by IHC, and the cut-off value was set to ≥1%. HR (hormone-receptor) positive was defined as ER or PR positive. The detection of *HER2* refers to the American Society of Clinical Oncology/College of American Pathologists (ASCO/CAP) guidelines for *HER2* testing. *HER2* negative breast cancer was divided into two groups: *HER2*-zero and *HER2*-low. *HER2*-zero was defined as IHC 0, and *HER2*-low was defined as IHC 1+ or IHC 2+/FISH negative.

In this study, the patients received palbociclib plus AI or fulvestrant therapy until disease progression, death or unacceptable toxicity. Palbociclib was given at a dose of 125mg orally as the initial dose once a day on d1 to d21 every four weeks. Anastrozole or fulvestrant was administered concurrently. Fulvestrant was administered intramuscularly at a dose of 500 mg on d1 and d15 of cycle 1 and then every four weeks from cycle 2. Anastrozole 1mg was administered orally on a continuous daily dosing schedule. For premenopausal patients, gonadotropin-releasing hormone (GnRH) agonist was administered before endocrine therapy. 3.6 mg of goserelin was injected subcutaneously once every 28 days. After treatment, imaging examinations were performed after every two cycles of in all patients to evaluate the clinical efficacy.

### Statistical analysis

All the statistical analyses were performed with SPSS 22.0 software (SPSS Inc., IL, US) software. P<0.05 was considered significant. Pearson’s chi squared test or Fisher’s exact test were used to compare the difference between groups. Survival curves of patients were estimated by the Kaplan-Meier method. The follow-up deadline is June 1, 2021. Progression free survival (PFS) was defined as from the date of palbociclib treatment to disease progression or death. Overall survival (OS) was defined as from the date of palbociclib treatment to patient death or last follow-up.

## Results

### Patient and treatment

A total of 45 patients were enrolled in the present study. **Table 1** shows patient and treatment characteristics. In the entire cohort, 73.3% patients were ECOG PS 0-1 and 26.7% patients were ECOG PS 2. The proportion of premenopausal patients is 64.4%, and 35.6% patients were postmenopausal. The disease status in 4(8.9%) patients was initial diagnosis stage IV, and in 41(91.1%) patients was recurrence and metastasis. Therefore, most patients in this study had received prior surgery (41,91.1%), neo-adjuvant/adjuvant chemotherapy (32,71.1%) and adjuvant endocrine therapy (39,86.7%).

**Table 1:** Patient and treatment characteristics.

Characteristic	Total (n = 45) n (%)	HER2-zero (n = 24) n (%)	HER2-low (n = 21) n (%)	*P*
**Age (years, median)**	50 (27-84)	43 (27-84)	54 (36-73)	
**ECOG**				0.280
0-1	33 (73.3)	16 (66.7)	17 (81.0)	
2	12 (26.7)	8 (33.3)	4 (19.0)	
**Menopausal status**				0.739
Premenopausal	29 (64.4)	16 (66.7)	13 (61.9)	
Postmenopausal	16 (35.6)	8 (33.3)	8 (38.1)	
**Prior chemotherapy before metastasis**				0.965
Neo-adjuvant/Adjuvant	32 (71.1)	17 (70.8)	15 (71.4)	
None	13 (28.9)	7 (29.2)	6 (28.6)	
**Prior adjuvant endocrine therapy**				0.691
Tamoxifen	21 (46.7)	10 (41.7)	11 (52.4)	
AI	18 (40.0)	10 (41.7)	8 (38.1)	
None	6 (13.3)	4 (16.7)	2 (9.5)	
**Prior surgery before metastasis**				0.889
Yes	41 (91.1)	22 (91.7)	19 (90.5)	
No	4 (8.9)	2 (8.3)	2 (9.5)	
**Disease status**				0.889
Initial diagnosis stage IV	4 (8.9)	2 (8.3)	2 (9.5)	
Recurrence and metastasis	41 (91.1)	22 (91.7)	19 (90.5)	
**HR status at metastasis stage**				0.718
ER+ and PR+	32 (71.1)	17 (70.8)	15 (71.4)	
ER+ and PR-	10 (22.2)	6 (25.0)	4 (19.0)	
ER- and PR+	3 (6.7)	1 (4.2)	2 (9.5)	
**Metastatic site**				0.432
Lymph node	17 (37.8)	9 (37.5)	8 (38.1)	
Chest wall	8 (17.8)	2 (8.3)	6 (28.6)	
Liver	11 (24.4)	7 (29.2)	4 (19.0)	
Lung	20 (44.4)	8 (33.3)	12 (57.1)	
Bone	29 (64.4)	17 (70.8)	12 (57.1)	
Brain	3 (6.7)	1 (4.2)	2 (9.5)	
**Number of metastatic sites**				0.565
1-2	28 (62.2)	14 (58.3)	14 (66.7)	
≥ 3	17 (37.8)	10 (41.7)	7 (33.3)	
**Metastatic sites type**				0.322
Visceral	31 (68.9)	15 (62.5)	16 (76.2)	
Non-Visceral	14 (31.1)	9 (37.5)	5 (23.8)	
**Prior chemotherapy after metastasis**				0.511
Yes	17 (37.8)	8 (33.3)	9 (42.9)	
No	28 (62.2)	16 (66.7)	12 (57.1)	
**Prior endocrine therapy after metastasis**				0.580
AI	23 (51.1)	14 (58.3)	9 (42.9)	
Fulvestrant	4 (8.9)	2 (8.3)	2 (9.5)	
None	22 (48.9)	10 (41.7)	12 (57.1)	
**Treatment manner**				0.632
CDK4/6 + AI	24 (53.3)	12 (50.0)	12 (57.1)	
CDK4/6 + Fulvestrant	21 (46.7)	12 (50.0)	9 (42.9)	
**Treatment line**				0.951
1	14 (31.1)	7 (29.2)	7 (33.3)	
2	18 (40.0)	10 (41.7)	8 (38.1)	
≥ 3	13 (28.9)	7 (29.2)	6 (28.6)	

ECOG, Eastern Cooperative Oncology Group; HR, hormone receptor; AI, aromatase inhibitor; CDK4/6, cyclin-dependent kinase 4 and 6.

The proportions of ER+/PR+, ER+/PR- and ER-/PR+ at metastasis stage were 71.1%, 22.2% and 6.7%, respectively. The common metastatic sites included bone (64.4%), lung (44.4%), lymph node (37.8%), liver (24.4%), chest wall (17.8%) and brain (6.7%). Number of metastatic sites in 28 (62.2%) patients were 1 or 2, and the other 17 (37.8%) patients were 3 or more. Thirty-one patients (68.9%) had visceral metastasis, and the other 14 patients (31.1%) had non-visceral metastasis. In metastatic disease stage, 37.8% patients had received chemotherapy and 51.1% patients had received endocrine therapy, including AI and fulvestrant. Palbociclib was administered as first line in 14 (31.1%) patients, as second line in 18 (40.0%) patients and as third or above line in 13 (28.9%) patients. 24 (53.3%) patients received palbociclib plus anastrozole and the other 21 (46.7%) patients received palbociclib plus fulvestrant.

In the the entire cohort, 24 (53.3%) patients were *HER2*-zero, and 21 (46.7%) patients were *HER2*-low. There were no statistically significant differences between *HER2*-zero and *HER2*-low breast cancer with respect to baseline clinicopathological characteristics.

### Efficacy

In the the entire cohort, complete response (CR) was not obtained, 16 patients had partial response (PR), 19 patients were evaluated as stable disease (SD) and 10 patients were evaluated as progressive disease (PD). The overall ORR and DCR were 35.6% (16/45) and 77.8% (35/45), respectively (**Table 2**). In *HER2*-zero breast cancer patients, CR was not obtained, 10 patients had PR, 9 patients were evaluated as SD and 5 patients were evaluated as PD. The overall ORR and DCR were 41.7% (10/24) and 79.2% (19/24), respectively. In *HER2*-low breast cancer patients, CR was not obtained, 6 patients had PR, 10 patients were evaluated as SD and 5 patients were evaluated as PD. The overall ORR and DCR were 28.6% (6/21) and 76.2% (16/21), respectively. There were no significant differences in ORR and DCR between *HER2*-zero and *HER2*-low breast cancer patients. Although there is no statistical difference, the ORR and DCR in patients who received palbociclib as third or above line therapy were inferior to patients who received palbociclib as first and second line therapy. There were also no statistically significant differences in ORR and DCR with respect to treatment manner (*CDK4/6* + AI vs. *CDK4/6* + Fulvestrant) and metastatic sites type (non-Visceral vs. visceral).

**Table 2:** Efficacy of CDK4/6 inhibitor treatment in metastatic breast cancer.

Parameter	Best response				ORR	*P*	DCR	*P*	Median PFS (95%CI)	*P*
	CR	PR	SD	PD						
**Total**	0	16	19	10	35.6%		77.8%		16.2(12.0-20.4)	
**HER2 status**						0.360		0.811		0.263
HER2-zero	0	10	9	5	41.7%		79.2%		16.2(10.9-21.5)	
HER2-low	0	6	10	5	28.6%		76.2%		14.1(9.0-19.2)	
**Treatment line**						0.196		0.589		**0.019**
First-line	0	6	6	2	42.9%		85.7%		22.6(19.3-25.9)	
Second-line	0	8	6	4	44.4%		77.8%		16.5(10.1-22.9)	
Third-line or above	0	2	7	4	15.4%		69.2%		10.8(9.3-12.3)	
**Treatment manner**						0.771		0.231		0.115
CDK4/6 + AI	0	9	8	7	37.5%		70.8%		10.8(2.9-18.7)	
CDK4/6 + Fulvestrant	0	7	11	3	33.3%		85.7%		16.9(10.1-23.7)	
**Metastatic sites type**						0.511		0.102		0.158
Non-Visceral	0	4	9	1	28.6%		92.9%		21.5(12.9-30.1)	
Visceral	0	12	10	9	38.7%		71.0%		12.9(6.3-19.5)	

CR, complete response; PR, partial response; SD, stable disease; PD, progressive disease; ORR, overall response rate; DCR, disease control rate; PFS, progression free survival; OS, overall survival. Bold values: P–0.05.

**Figure 1:**
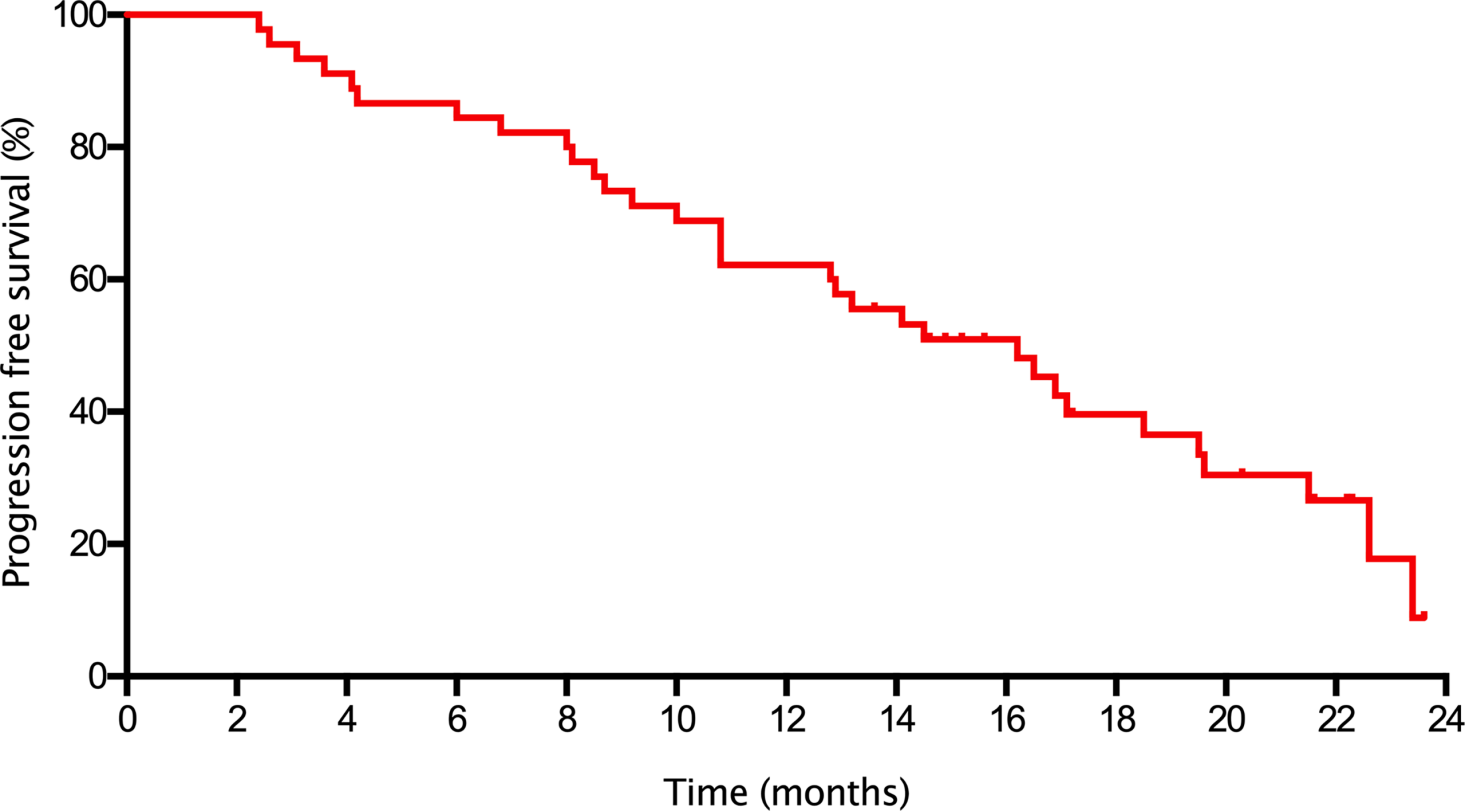
Kaplan-Meier curve of PFS in the entire cohort.

**Figure 2:**
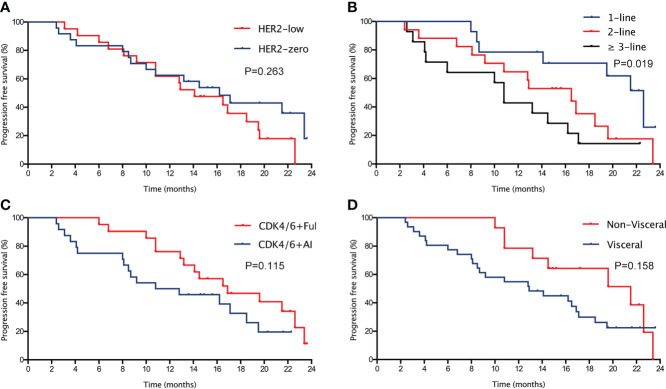
Kaplan-Meier curve of PFS **(A)** in the entire cohort with different HER2 status (HER2-zero vs. HER2-low). Kaplan-Meier curve of PFS **(B)** in patients with different treatment line (first-line, second-line vs. third-line or above). Kaplan-Meier curve of PFS **(C)** in patients with different treatment manner (CDK4/6 + AI vs. CDK4/6 + fulvestrant). Kaplan-Meier curve of PFS **(D)** in patients with different metastatic sites type (non-visceral vs. visceral).

In the the entire cohort, the median PFS was 16.2 (95% CI= 12.0-20.4) months (**Figure 1**) and the median OS was not reached. The median PFS were 16.2 (95%CI=10.9-21.5) and 14.1 (95%CI=9.0-19.2) months in the *HER2*-zero and *HER2*-low breast cancer patients, respectively (P=0.263; **Figure 2A**). The median PFS in patients who received palbociclib as third or above line therapy was 10.8 (95%CI=9.3-12.3) months, which was worse than patients who received palbociclib as first [22.6 (95%CI=19.3-25.9) months] and second [16.5 (95%CI=10.1-22.9) months] line therapy (P=0.019; **Figure 2B**). There were also no statistically significant differences in median PFS with respect to treatment manner (*CDK4/6* + AI vs. *CDK4/6* + Fulvestrant) and metastatic sites type (non-Visceral vs. visceral) (**Figures 2C, D**).

### Safety

The spectrum of treatment-related toxicity was consistent with previous clinical studies. Treatment-related death and unexpected side effects were not occurred (**Table 3**). Treatment was temporarily interrupted in 28 (62.2%) patients as a result of adverse events (AEs), and 8 (17.8%) patients underwent dose reductions due to grade 3-4 AEs. The most common treatment-related AEs was hematological toxicity, including neutropenia (38, 84.4%), leukopenia (22, 48.9%), decreased platelet (11, 24.4%) and anemia (9, 20.0%). The common non-hematological toxicity were fatigue (19, 42.2%), asthenia (16, 35.6%), rash (8, 17.8%), constipation (8, 17.8%), anorexia (7, 15.6%), alopecia (7, 15.6%), nausea or vomiting (6, 13.4%) and diarrhea (5, 11.1%). The incidence of grade 3-4 AEs was 57.8%, including neutropenia, leukopenia, increased alanine aminotransferase (ALT)/aspartate aminotransferase (AST) and diarrhea.

**Table 3:** Treatment-related toxicity.

Adverse Event	All			HER2-zero			HER2-low	
	All Grade	≥ Grade3		All Grade	≥ Grade3		All Grade	≥ Grade3
**Non-hematologic**								
Alopecia	7 (15.6)	0		3 (12.5)	0		4 (19.0)	0
Fatigue	19 (42.2)	0		10 (41.7)	0		9 (42.9)	0
Nausea or Vomiting	6 (13.3)	0		3 (12.5)	0		3 (14.3)	0
Anorexia	7 (15.6)	0		4 (16.7)	0		3 (14.3)	0
Diarrhea	5 (11.1)	1 (2.2)		3 (12.5)	1 (4.2)		2 (9.5)	0
Muscle pain/joint pain	4 (8.9)	0		2 (8.3)	0		2 (9.5)	0
Constipation	8 (17.8)	0		4 (16.7)	0		4 (19.0)	0
Pyrexia	4 (8.9)	0		3 (12.5)	0		1 (4.8)	0
Rash	8 (17.8)	0		4 (16.7)	0		4 (19.0)	0
Oral mucositis	3 (6.7)	0		2 (8.3)	0		1 (4.8)	0
Asthenia	16 (35.6)	0		8 (33.3)	0		8 (38.1)	0
**Hematologic**								
Leukopenia	22 (48.9)	9 (20.0)		11 (45.8)	5 (20.8)		11(52.4)	4(19.0)
Neutropenia	38 (84.4)	25 (55.6)		20 (83.3)	13 (54.2)		18 (85.7)	12 (57.1)
Anemia	9 (20.0)	0		4 (16.7)	0		5 (23.8)	0
Decreased platelet	11 (24.4)	0		5 (20.8)	0		6 (28.6)	0
Increased ALT/AST	5 (11.1)	1 (2.2)		3 (12.5)	0		2 (9.5)	1 (4.8)

The number of patients with interruptions and dose reduction as a result of AEs in *HER2*-zero breast cancer were 15(62.5%) and 4(16.7%), while in *HER2*-low breast cancer were 13(61.9%) and 4(19.0%), respectively. The incidence of grade 3-4 AEs in these two groups were 58.3% and 57.1%, respectively. These were no statistically significant differences in treatment-related toxicity among breast cancer patients with *HER2*-zero and *HER2*-low.

## Discussion

The present study compared the clinical efficacy of *CDK4/6* inhibitor for HR positive patients with *HER2*-zero or *HER2*-low metastatic breast cancer, no significant differences were observed in treatment response and median PFS between the two groups. To our knowledge, until now this is the first study of evaluating whether *HER2*-low expression will affect the clinical efficacy of *CDK4/6* inhibitor for patients with HR+/*HER2*- metastatic breast cancer based on real-world clinical practice. Our present study demonstrated that *HER2*-low expression does not affect the clinical efficacy of palbociclib, which did not support incorporating *HER2*-low into systemic therapy decisions for patients with HR+/*HER2*- metastatic breast cancer treated with *CDK4/6* inhibitor.

In breast cancer, about 15%-20% of patients have abnormal amplification or overexpression of *HER2* gene (16). *HER2* status has become an important indicator for molecular typing of breast cancer, and simultaneously, *HER2* is also an important target for breast cancer (17). The clinical application of anti *HER2* targeted drugs represented by trastuzumab in neoadjuvant therapy, adjuvant therapy and advanced rescue therapy has changed the diagnosis and treatment mode of breast cancer, and also greatly improved the prognosis of *HER2* positive breast cancer patients (18–20). However, the expression level of *HER2* was a continuous process in pathology, rather than the concept of absolute distinction between positive and negative (21, 22). Therefore, the concept of *HER2* low expression emerged (23). Most clinical trials use IHC 1+, or IHC 2+ and FISH negative as the definition of *HER2*-low breast cancer. The proportion of breast cancer with *HER2* low expression is as high as 45%-55% according to this criteria. So far, little is currently known about the biological behavior of tumors with *HER2* low expression.

There is still controversy over *HER2*-low as a distinct biological subtype of breast cancer. Several previous studies suggested that the proportion of *HER2*-low in HR positive breast cancer was higher than in triple negative breast cancer (TNBC) (24). Data from our institution demonstrated that in HR positive breast cancer, *HER2*-low patients showed less lymph node metastatic burden and earlier stage. In contrast, *HER2*-low breast cancer had later clinical stage than *HER2*-zero patients in TNBC. Another study suggested that histological grade 2 was more common in patients with *HER2*-low breast cancer, and the expression level of *Ki67* was significantly lower than that in patients with *HER2*-zero *(* 25). PAM50 gene analysis showed that in HR+ breast cancer, *HER2*-low breast cancer had more expressed *ERBB2* and luminal-related genes than *HER2*-zero breast cancer, but not in TNBC (26). The genomic data from 523 breast cancer patients suggested that *HER2*-low and *HER2*-zero breast cancer exhibit distinct gene mutation signatures (27). HER2-zero tumors had more gene mutations in *p53* signaling and cell cycle pathway, and *HER2*-low breast cancer exhibit more gene mutations which involved in *PI3K-Akt* signaling pathway. However, in metastatic breast cancer, whether *HER2*-low and *HER2*-zero breast cancer differ in clinical and gene expression features has not been reported. In our present study, no significant differences were found between *HER2*-zero and *HER2*-low metastatic breast cancer in clinical and pathological characteristics.

Low expression of *HER2* may be related to treatment efficacy of breast cancer. In neoadjuvant chemotherapy (NAC) setting, a previous pooled analysis of four neoadjuvant chemotherapy clinical trials (GeparSepto, GeparOcto, GeparX, and Gain-2 neoadjuvant) confirmed that *HER2*-low breast cancer had a significantly lower pathological complete response(pCR) rate than *HER2*-zero patients (24). Our recent study analyzed 314 *HER2* negative breast cancer patients who received NAC and found that with the pCR defined as ypT0ypN0, the pCR rate in *HER2*-low breast cancer was significantly lower than *HER2*-zero breast cancer in entire cohort(24.3% vs. 36.4%, P=0.032) and HR-positive subgroup(18.7% vs. 32.1%, P=0.035). In targeted anti-*HER2* therapy for metastatic breast cancer setting, DS8201-A-J101, DESTINY-Breast04 and DESTINY-Breast06 studies showed that trastuzumab deruxtecan (T-DXd, DS-8201a) provide a novel treatment strategy for patients with low *HER2* expression (11, 12). In endocrine therapy for metastatic breast cancer setting, our present study compared the clinical efficacy of *CDK4/6* inhibitor for patients with HR positive *HER2*-zero and *HER2*-low metastatic breast cancer, no significant differences were observed in treatment response and patient prognosis. In this study, we show for the first time that *HER2*-low expression does not affect the clinical outcomes of metastatic breast cancer treated with *CDK4/6* inhibitor.

Treatment-related toxicity of palbociclib was consistent with previous relevant clinical trials, hematological toxicity was the most common AEs (28–30). No statistically significant differences in treatment-related toxicity between *HER2*-zero and *HER2*-low breast cancer patients were found, including the incidence of grade 3-4 AEs, interruptions and dose reduction.

Our present study has several limitations, because it is a single center and observational study, and the number of patients included is not many. Therefore, the results of this study need future validation clinical trials to confirm whether *HER2*-low expression will affect the treatment decision of metastatic breast cancer.

## Conclusion

In conclusion, these data confirm that *HER2*-low expression does not affect the clinical efficacy of palbociclib and our present study did not support incorporating HER2-low into systemic therapy decisions for patients with HR+/*HER2*- metastatic breast cancer treated with *CDK4/6* inhibitor.

## Data availability statement

The raw data supporting the conclusions of this article will be made available by the authors, without undue reservation.

## Ethics statement

The studies involving human participants were reviewed and approved by The ethics committee of the Henan Provincial People’s Hospital. The patients/participants provided their written informed consent to participate in this study.

## Author contributions

All authors contributed to the article and approved the submitted version. YS and HL designed the research, analyzed the data and drafted the paper. YS, ZL, YY, and QC were mainly responsible for data collection and analysis. YH, CL, BN and FZ were primarily responsible for statistical analysis.

## Funding

This work was supported by Beijing Medical Award Foundation Project (No.YXJL-2020-0941-0748) and Medical Science and Technique Foundation of Henan Province (No. LHGJ20210055).

## Conflict of interest

The authors declare that the research was conducted in the absence of any commercial or financial relationships that could be construed as a potential conflict of interest.

## Publisher’s note

All claims expressed in this article are solely those of the authors and do not necessarily represent those of their affiliated organizations, or those of the publisher, the editors and the reviewers. Any product that may be evaluated in this article, or claim that may be made by its manufacturer, is not guaranteed or endorsed by the publisher.
